# Case Report: Stepwise Anti-Inflammatory and Anti-SARS-CoV-2 Effects Following Convalescent Plasma Therapy With Full Clinical Recovery

**DOI:** 10.3389/fimmu.2021.613502

**Published:** 2021-04-21

**Authors:** Aurelia Zimmerli, Matteo Monti, Craig Fenwick, Isabella Eckerle, Catherine Beigelman-Aubry, Céline Pellaton, Katia Jaton, Dominique Dumas, Gian-Marco Stamm, Laura Infanti, Heidrun Andreu-Ullrich, Daphné Germann, Marie Mean, Peter Vollenweider, Raphael Stadelmann, Maura Prella, Denis Comte, Benoit Guery, David Gachoud, Nathalie Rufer

**Affiliations:** ^1^Department of Internal Medicine, Lausanne University Hospital and University of Lausanne, Lausanne, Switzerland; ^2^Medical Education Unit, School of Medicine, Faculty of Biology and Medicine, University of Lausanne, Lausanne, Switzerland; ^3^Division of Immunology and Allergy, Department of Medicine, Lausanne University Hospital and University of Lausanne, Lausanne, Switzerland; ^4^Laboratory of Virology and Geneva Centre for Emerging Viral Diseases, Geneva University Hospitals and Faculty of Medicine, University of Geneva, Geneva, Switzerland; ^5^Department of Radiology and Interventional Radiology, Lausanne University Hospital and University of Lausanne, Lausanne, Switzerland; ^6^Institute of Microbiology, Lausanne University Hospital and University of Lausanne, Lausanne, Switzerland; ^7^Regional Blood Transfusion Service, Swiss Red Cross (SRC), Basel, Switzerland; ^8^Interregional Blood Transfusion SRC, Epalinges, Switzerland; ^9^Department of Geriatric Medicine and Geriatric Rehabilitation, Lausanne University Hospital, Lausanne, Switzerland; ^10^Division of Hematology, Department of Oncology, Lausanne University Hospital and University of Lausanne, Lausanne, Switzerland; ^11^Department of Respiratory Disease, Lausanne University Hospital and University of Lausanne, Lausanne, Switzerland; ^12^Division of Infectious Diseases, Department of Medicine, Lausanne University Hospital and University of Lausanne, Lausanne, Switzerland; ^13^Department of Oncology, Lausanne University Hospital and University of Lausanne, Epalinges, Switzerland

**Keywords:** chronic SARS-CoV-2 infection, severe immunosuppression, B-cell depletion, convalescent plasma therapy, neutralizing antibodies, viral clearance

## Abstract

In these times of COVID-19 pandemic, concern has been raised about the potential effects of SARS-CoV-2 infection on immunocompromised patients, particularly on those receiving B-cell depleting agents and having therefore a severely depressed humoral response. Convalescent plasma can be a therapeutic option for these patients. Understanding the underlying mechanisms of convalescent plasma is crucial to optimize such therapeutic approach. Here, we describe a COVID-19 patient who was deeply immunosuppressed following rituximab (anti-CD20 monoclonal antibody) and concomitant chemotherapy for chronic lymphoid leukemia. His long-term severe T and B cell lymphopenia allowed to evaluate the treatment effects of convalescent plasma. Therapeutic outcome was monitored at the clinical, biological and radiological level. Moreover, anti-SARS-CoV-2 antibody titers (IgM, IgG and IgA) and neutralizing activity were assessed over time before and after plasma transfusions, alongside to SARS-CoV-2 RNA quantification and virus isolation from the upper respiratory tract. Already after the first cycle of plasma transfusion, the patient experienced rapid improvement of pneumonia, inflammation and blood cell counts, which may be related to the immunomodulatory properties of plasma. Subsequently, the cumulative increase in anti-SARS-CoV-2 neutralizing antibodies due to the three additional plasma transfusions was associated with progressive and finally complete viral clearance, resulting in full clinical recovery. In this case-report, administration of convalescent plasma revealed a stepwise effect with an initial and rapid anti-inflammatory activity followed by the progressive SARS-CoV-2 clearance. These data have potential implications for a more extended use of convalescent plasma and future monoclonal antibodies in the treatment of immunosuppressed COVID-19 patients.

## Introduction

COVID-19 is a rapidly evolving novel infectious disease caused by SARS-CoV-2 (severe acute respiratory syndrome coronavirus 2), leading to substantial morbidity and mortality. Due to its worldwide spread, immunocompromised patients, particularly those receiving B-cell depleting agents and who are unable to mount a specific humoral response to SARS-CoV2, may be at particular risk for a severe COVID-19 course (1, 2). In fact, repeated administration of rituximab (an anti-CD20 monoclonal antibody), is largely applied in the treatment of various B-cell malignancies and may lead to prolonged B-cell depletion and impaired production of total immunoglobulins (Ig) G and IgM (3, 4). Plasma provided by convalescent donors (i.e. convalescent plasma) has been proposed as a therapeutic option for COVID-19 (5), based on its partial effectiveness in other respiratory infections (6, 7). Consistent with this, several clinical trials have shown a benefit of convalescent plasma in COVID-19 patients with severe respiratory disease (8–10). It is likely that high anti-SARS-CoV2 IgG antibody levels (9, 10) are important for therapeutic effects. However, convalescent plasma may have additional and currently still unknown mechanisms of action (11).

This report presents the successful treatment of a severely immunocompromised patient with prolonged COVID-19 by four cycles of convalescent plasma transfusion. Our study provides insight into at least two distinct modes of action of plasma components. The first one may be related to its proposed anti-inflammatory activity. The second mechanism is mediated by SARS-CoV-2-specific IgG and neutralizing antibodies present in convalescent immune plasma, associated with progressive viral clearance, resulting in full clinical recovery from infection. Application of convalescent plasma with relative high titers of anti-SARS-CoV-2 antibodies represents a promising approach in the context of immunosuppressed patients with prolonged COVID-19 disease.

## Case Report

**Figure 1:**
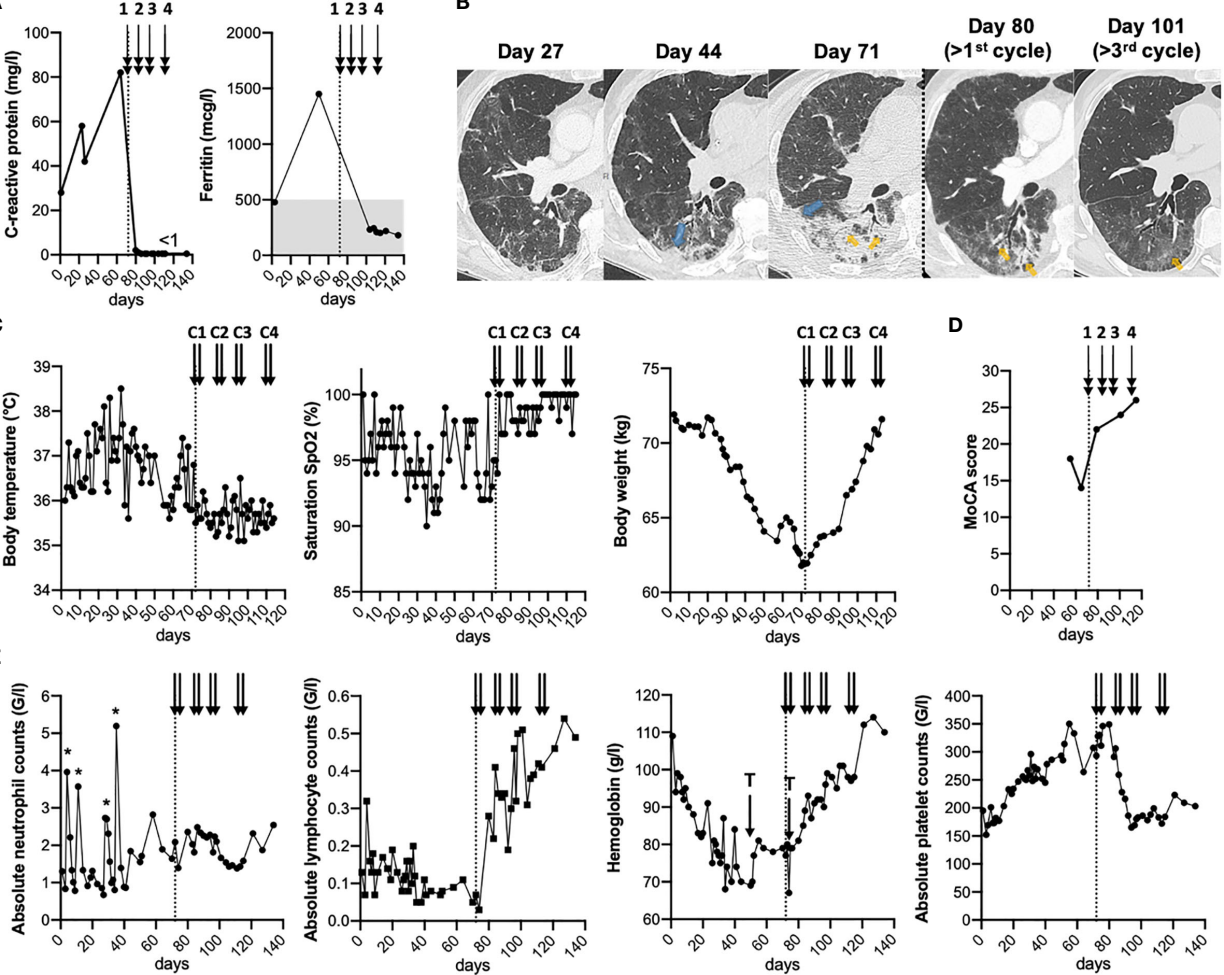
Clinical, biological and radiological follow-up before and after plasma transfusions. **(A–E)**, Timeline of chronic SARS-COV-2 infection in a severely immunosuppressed patient showing inflammatory markers **(A)**, chest CT scan **(B)**, clinical parameters **(C)**, MoCA (Montreal Cognitive Assessment) scores **(D)**, and complete blood counts **(E)**. **(B)**, Axial slices focused at the level of the apical segment of the right lower lobe. Progressive worsening from multiple ill-defined areas of ground glass opacities progressing to patchy alveolar consolidation of increasing size. Note the subpleural (blue arrows) and peribronchial (orange arrows) distribution of the lesions with progressive improvement on post-therapeutic follow-up, alveolar consolidation being replaced by ground glass opacity. **(A–E)**, Arrows indicate the 4 cycles of plasma transfusion (two units given on two consecutive days of each cycle). The “*” show the three injections of granulocyte-colony stimulating factor (filgrastim), while “T” indicates the two red blood cell transfusions. Of note, filgrastim injections (< day 40) and red blood cell transfusions did not result in symptom improvements.

A 74-year-old man, in complete remission of a chronic lymphoid leukemia (CLL) after 6 cycles of rituximab and bendamustin (last therapy administrated in December 2019), presented to our emergency unit on April 1^st^ 2020 with asthenia, loss of weight, dry cough and diarrhea since a month. He was otherwise healthy with well-controlled arterial hypertension and type 2 diabetes mellitus. SARS-CoV-2 RNA was detected (7x10^6^ copies/ml) (12, 13) from a nasopharyngeal swab (defined as day 1). Laboratory analyses showed a moderate neutropenia, but severe T (114 cell/mm^3^) and B (1 cell/mm^3^) lymphopenia with reduced total immunoglobulin (Ig) G and IgM levels (**Supplementary Figure 1A**), while inflammatory markers (C-reactive protein and ferritin) were elevated (**Figure 1A**). Chest computed tomography (CT) revealed bilateral multifocal subpleural and peribronchial ground-glass opacities typical of COVID-19 pneumonia (14) (**Figure 1B** and **Supplementary Figure 2**). The clinical condition gradually deteriorated with sub-febrile episodes, persisting dry cough and diarrhea, and progressive weight loss and cognitive dysfunction (**Figures 1C, D** and **Supplementary Information**). Inflammatory parameters and blood cell counts remained abnormal (**Figures 1A, E**), while persisting SARS-CoV-2 infection was confirmed (**Supplementary Figure 1B**). Complementary investigations excluded other diagnoses (**Supplementary Information**). No specific antiviral agents were introduced given the mild symptoms of COVID-19 (e.g. absence of hypoxemia).

In summary, the patient developed a long-lasting SARS-CoV-2 infection likely related to his severe immunosuppressive status. Consequently, we hypothesized that convalescent plasma could be beneficial in this particular case, by providing virus-specific neutralizing antibodies as well as a potential anti-inflammatory effect. Plasma units (3x200 ml/donor) were obtained from three selected donors, who had fully recovered from mild COVID-19 disease (**Supplementary Information**). Each donor presented relatively high IgG antibody titers against the S1 (spike)-protein using Euroimmun ELISA (15) (**Supplementary Figure 3A**). The first cycle of ABO-compatible plasma transfusion (two units on two consecutive days) was given on days 72 and 73 after diagnosis of SARS-CoV-2 infection, followed by three additional cycles, administered 10 to 15 days apart (**Supplementary Figure 3A**).

## Results

### Rapid Improvement of Clinical, Inflammatory and Radiological Parameters Following Plasma Transfusion

**Figure 2:**
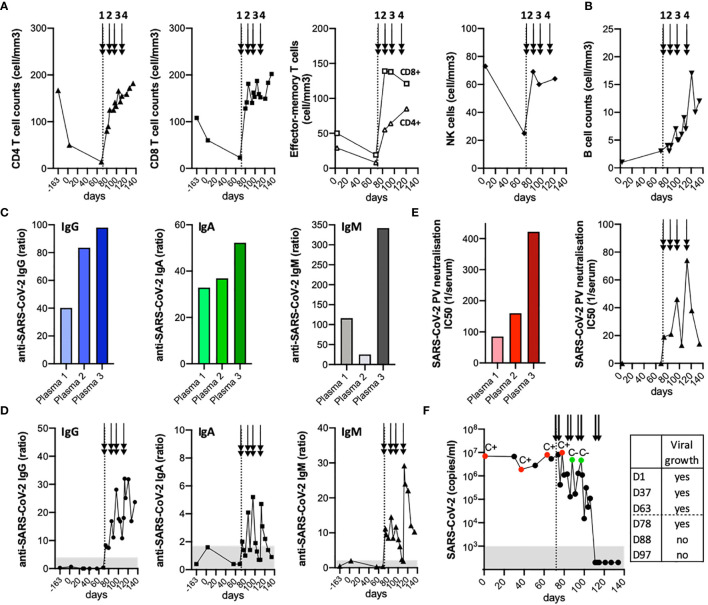
Anti-SARS-CoV-2 antibody and SARS-CoV-2 mRNA follow-up before and after plasma transfusions. **(A, B)**, Timeline showing absolute CD4 and CD8 T cell counts, including effector-memory subsets and NK cell counts **(A)**, and absolute B cell counts **(B)**. **(C, D)**, Anti-SARS-CoV-2 S protein IgG, IgA and IgM antibody levels as assessed by an in-house developed Luminex assay for each plasma **(C)** as well as in the patient’s serum before and following plasma transfusions **(D)**. **(E)**, Activity of neutralizing antibodies was assessed by a SARS-CoV-2 pseudovirus neutralization assay for each plasma and in patient’s serum at different time-points. **(F)**, Over-time follow-up of SARS-CoV-2 RNA detection in nasopharyngeal swabs. The cytopathic effect on VeroE6 cells was evaluated after inoculation with SARS-CoV-2 from nasopharyngeal swabs: C+, successful virus isolation; C-, absence of virus isolation. **(A–F)**, The arrows indicate the 4 cycles of plasma transfusion (two units given on two consecutive days of each cycle).

Within the first eight days after the start of plasma transfusions, the patient improved clinically, biologically and radiologically (**Figure 1**). We observed a rapid normalization of the C-reactive protein, while absolute platelet counts and hemoglobin levels showed a more gradual return-to-normal. Follow-up chest CT scan confirmed a significant improvement in pulmonary infiltrates (**Figure 1B** and **Supplementary Figure 2**). Absolute lymphocyte counts also substantially improved (**Figure 2A**), and largely consisted of increased levels of memory-effector CD4 and CD8 T cells and of NK cells (by CyTOF multi-parametric mass cytometry (16); **Supplementary Figure 4**). Only a moderate rise was observed for total B cell counts (**Figure 2B**), mostly composed of unswitched memory B cells at day 121 (16); **Supplementary Figure 4F**), while total IgM and IgG remained globally stable (**Supplementary Figure 1A**). These data indicate clear improvements of inflammation, pneumonia and blood cell counts, already after the 1^st^ cycle of convalescent plasma transfusion.

### Cumulative Increase in Anti-SARS-CoV-2 IgG Titers Following Successive Plasma Transfusions

Most patients with COVID-19 develop SARS-CoV-2 IgM and IgG antibody responses within 19 days after symptom onset (17). To investigate whether such antibodies were transferred to our patient following successive cycles of plasma transfusion, we monitored the anti-SARS-CoV-2 S protein-specific IgG, IgA and IgM antibody levels by an in-house developed Luminex assay (18) (**Supplementary Information**). There was a remarkable heterogeneity among the three plasma donors, with plasma/donor 3 exhibiting highest levels of specific antibodies (**Figure 2C** and **Supplementary Figure 3B**). Whereas no anti-S IgG response was detected in the patient’s serum before the start of plasma transfusion, the antibody titers increased progressively, up to 30-fold over the baseline reference after the 4^th^ cycle (**Figure 2D** and **Supplementary Figure 3B**). A similar trend, albeit at much lower levels, was found for anti-S IgA antibodies. Instead, anti-S IgM antibodies revealed two peaks following serial plasma transfusions (**Figure 2D**), again related to the level of specific-IgM antibodies of each plasma donor, with plasma/donor 3 showing highest titers (**Figure 2C**).

### Temporal Association Between Detection of SARS-CoV-2 Neutralizing Antibodies and Viral Clearance

The activity of neutralizing antibodies was next measured by a SARS-CoV-2 pseudovirus neutralization assay in all three plasma as well as in patient’s serum before and after plasma transfusions (**Supplementary Information**; **Supplementary Figure 3C**). We further observed a boost of SARS-CoV-2 neutralizing activity after each cycle of plasma administration, in line with the higher antibody activity found in plasma/donor 3 when compared to the two others (**Figure 2E**). Importantly, this increase in SARS-CoV-2 neutralizing antibodies (**Figure 2E**) was inversely associated to the gradual decline observed in viral loads in nasopharyngeal swabs (**Figure 2F**), becoming undetectable for both E and RdRP genes (13) by day 111 of diagnosis.

To assess whether shedding of infectious SARS-CoV-2 still occurred after the start of plasma transfusions, we measured the presence of cultivable SARS-CoV-2 at different time-points (19) (**Supplementary Information**). While infectious virus could be isolated from nasopharyngeal swabs prior and following the 1^st^ cycle of convalescent plasma transfusion, this was no longer the case upon the 2^nd^ cycle (**Figure 2F**). Finally, the patient received one dose of intravenous immunoglobulin (IVIg; 0.4 g/kg) on day 114, providing additional passive immune protection against common pathogens, before being discharged from hospital (on day 115). He was considered as cured after two weeks of consecutive negative swabs (on day 127), but remaining potentially vulnerable to SARS-CoV-2 re-infection. The patient was followed initially by weekly and then monthly outpatient care. During this period, we observed an initial decline in anti-S IgG titers (18), followed by their subsequent stabilization over time (**Supplementary Figure 3D**). This was coincident to immune reconstitution, with the normalization of total B cell counts (**Supplementary Figure 3D**) and the appearance of naive B cells (16) (at day 203; **Supplementary Figure 4F**).

## Discussion

Collectively, our study offers novel evidence for a clear benefit of convalescent plasma in this particular case of COVID-19 disease, with the resolution of clinical, inflammatory and radiological parameters (**Figure 1**). Improvement of inflammation, pneumonia and blood cell counts preceded viral clearance. Importantly, this patient showed a temporal association between detection of SARS-CoV-2 neutralizing antibodies and virus clearance following successive cycles of plasma transfusion (**Figure 2**). Thus, our data provide insight into at least two distinct modes of action of plasma components. The first one is related to its proposed anti-inflammatory activity, similar to IVIg, widely used at high-dose for the treatment of several autoimmune diseases (11, 20). In this line, convalescent plasma therapy may help in modulating the immune response *via* F(ab’)_2_-dependent mechanisms including blockade of cell-cell interactions (via cell-surface receptors) and neutralization of cytokines, complement and autoantibodies (by anti-idiotypic antibodies). In addition, convalescent plasma activity might involve Fc-dependent pathways (e.g. modulation of Fcγ receptors on innate immune effector cells and B cells) (11, 20). A similar rapid anti-inflammatory effect was reported in a series of 17 consecutive patients with profound B-cell lymphopenia and prolonged COVID-19 infection, who were treated with 4 units of convalescent plasma (21). Specifically, Hueso and colleagues (21) observed a decrease in temperature and inflammatory parameters within a week, associated to oxygen weaning, while SARS-CoV-2 RNAemia decline occurred later, between 7 to 14 days after plasma transfusion.

Our observations support a second mechanism of action by SARS-CoV-2-specific IgG (18) and neutralizing antibodies present in convalescent immune plasma (**Figure 2**), which may mediate direct virus neutralization or other antibody-mediated pathways (e.g. complement activation, antibody-dependent cellular cytotoxicity). Passive antibody therapy has the great advantage to confer immediate immunity to vulnerable individuals (22). Interestingly, our data further suggest a relatively rapid effect of convalescent plasma with disappearance of cultivable SARS-CoV-2 readily after the second cycle of transfusion, despite intermediate levels of neutralizing antibodies and high RNA-positivity by qRT-PCR (**Figure 2**). This is in line with findings showing that neutralizing antibodies derived from COVID-19 patients may be effective, even at concentrations of 9 ng/ml or less (23). Strikingly, successive cycles of plasma transfusion (every 10-15 days) led to the cumulative increase in anti-S IgG titers, associated with a boost of neutralizing antibodies after each administration. Anti-S-specific IgM titers also followed a kinetic pattern related to the antibody levels of each transfused donor-plasma unit, indicating that a *de novo* endogenous antibody response was improbable at this stage. These observations are further in agreement with a recent report showing clinical resolution of COVID-19 following transfusion of convalescent plasma of high-titer neutralizing antibodies, in another case of humoral immunodeficiency (24). Finally, it is certainly possible that CD4 and CD8 T cells played critical roles (25) in the recovery of this patient who was primarily B cell deficient. This view is supported by reported cases of agammaglobulinemia patients who only presented mild COVID-19 disease (26).

## Conclusion

Convalescent immune plasma therapy revealed stepwise anti-inflammatory and anti-SARS-CoV-2 effects, resulting in full clinical recovery from infection in a severely ill immunocompromised patient. Our data are in line with other studies (21, 24, 27, 28) reporting symptom resolution and clinical improvement following passive transfer of anti-SARS-CoV-2 antibodies through convalescent plasma in patients with immunodeficiency. Together, this therapeutic option can be considered as safe and represents a promising approach in the context of immunosuppressed patients with prolonged COVID-19 disease. At present, it remains to be seen how this compares to future monoclonal anti-SARS-CoV-2 antibodies and other novel COVID-19 therapies.

## Data Availability Statement

The original contributions presented in the study are included in the article/**Supplementary Material**. Further inquiries can be directed to the corresponding authors.

## Ethics Statement

Ethical review and approval was not required for the study on human participants in accordance with the local legislation and institutional requirements. The patient/participants provided their written informed consent to participate in this study.

## Author Contributions

Study design and supervision: MMo, BG, DGa and NR. Patient care and acquisition of data: AZ, MMo, CB-A, DD, G-MS, DGe, MMe, PV, RS, MP, DC, BG and DGa. Development of methodology: CF, IE, CP, LI, and HA-U. Analysis and interpretation of data: AZ, CF, IE, CB-A, CP, KJ, DC, DGa and NR. Writing of the manuscript: AZ, DGa and NR. All authors contributed to the article and approved the submitted version.

## Funding

This study was supported by the Lausanne University Hospital and University of Lausanne (Lausanne, Switzerland), and Interregional Blood Transfusion SRC (Lausanne, Switzerland).

## Supplementary Information

Complementary clinical and methodological information for this case report can be found in the **Supplementary Information**.

## Conflict of Interest

The authors declare that the research was conducted in the absence of any commercial or financial relationships that could be construed as a potential conflict of interest.
