# Supplementary material for: Case Report: Stepwise Anti-Inflammatory and Anti-SARS-CoV-2 Effects Following Convalescent Plasma Therapy With Full Clinical Recovery
Source: Front Immunol. 2021 Apr 21;12:613502. doi: 10.3389/fimmu.2021.613502 (PMC8097002; doi:10.3389/fimmu.2021.613502)
Supplement: Supplementary file 1 [file DataSheet_1.pdf]

SUPPLEMENTARY MATERIAL

SUPPLEMENTARY CLINICAL INFORMATION

From day 15 onwards, the patient developed progressive cognitive dysfunction manifested as bradypsychia, disorientation, and short-term memory loss, contributing to reduced autonomy and falls. On day 55 after diagnosis, the Confusion Assessment Method (CAM) became positive in the absence of any cause other than the underlying infection. A brain Magnetic Resonance Imaging (MRI) did not show signs for ischemic stroke or encephalitis. The patient declined a lumbar puncture. A bone marrow biopsy and a PET-CT scan further excluded significant CLL progression (with a minimal residual disease of 1.8%) or other oncological occurrences. Microbiological culture and PCR following bronchoalveolar lavage were negative for atypical mycobacterial as well as for viral or parasitic opportunist infections, except for SARS-CoV-2 (**Supplementary Figure 1**).

SUPPLEMENTARY METHODS

Nasopharyngeal swabs analysis

Nasopharyngeal swabs were processed under airborne precaution. The SARS-CoV-2 RNA was detected by reverse transcription and real-time PCR assays using a fully automated molecular diagnostic platform MDx platform as described in (1). Two independent primers targeting RNA polymerase (RdRP) and envelope (E) genes were used as reported in (2, 3). All obtained Ct values were converted to viral loads based on plasmids positive controls, as reported in (3).

Convalescent donor plasma and transfusion

Convalescent plasma was collected from three male donors (i.e. plasma 1 to 3), eligible for blood donation according to the requirements of the Blood Donation Service, Swiss Red Cross, who had fully recovered for at least 28 days after COVID-19 onset and had relatively high titers of anti-SARS-CoV-2 IgG (anti-S1 protein, [4.43-7.67 S/CO], by ELISA from EuroImmun, (4, 5)). Of note, due to the limitation of available donors with an AB blood group, donor 1 and 2 were first selected at the Regional Blood Transfusion Service (Basel, Switzerland), while donor 3 was identified later on at the Interregional Blood Transfusion SRC (Vaud, Switzerland). Apheresis was performed using the Aurora™ (Fresenius Kabi) or the Trima Accel® (Terumo BCT) with the collection of 650 ml, sufficient for three units. Manufacturing occurred under good manufacturing practice (GMP) conditions, according to the current regulations (Swisstransfusion CRS and Swiss Federal Act on Medical Products). After collection and before splitting into single units, the leukocyte-depleted plasma was treated for pathogen-inactivation using amotosalen and UVA irradiation (INTERCEPT® system, Cerus Corporation). The plasma was further separated into three units (200+/-20 ml each) and kept frozen at -25°C.

Patient A.P. was eligible for the study and received four cycles of ABO-identical convalescent plasma transfusion (i.e. two plasma units of 200 ml, corresponding to 3 ml/kg/unit, on two consecutive days of each cycle) obtained from three selected donors, at an interval of 10 to 15 days between the transfusion cycles (**Supplementary Figure 3**). Each transfusion was administrated over a 45-minute period, without any adverse events.

Luminex anti-SARS-CoV-2 S protein IgG, IgA and IgM binding assay

Preparation of the Luminex beads coupled with the S protein trimer was performed as described in (6). S protein-coupled Luminex beads were added to Bio-Plex Pro 96-well flat bottom plates, washed with PBS, before adding 50 µl of a 1/300 dilution of individual serum (from the patient) or plasma (from the donors) to the wells (6). Plates were agitated at 500 rpm for 60 minutes on a plate shaker. Beads were then washed and anti-human IgG-PE or anti-human IgA-PE (OneLambda ThermoFisher) or anti-human IgM secondary antibody (BioConcept) was added. Plates were agitated for 45 minutes and washed again. Beads were resuspended in 80 µl of reading buffer and read directly on a Luminex FLEXMAP 3D plate reader (ThermoFisher). MFI signal of each test serum or plasma sample was divided by the mean signal for the negative control samples yielding a MFI ratio (6).

Quantitative analysis of serum or plasma levels of anti-S protein IgG antibodies was determined using MFI signal from 1/300, 1/900, 1/2700 and 1/81000 dilutions of sample compared to a standard curve generated with a purified anti-SARS-CoV-2 S protein monoclonal antibody (Clone CR3022; Lucerna Chem).

SARS-CoV-2 pseudovirus neutralization assay

A single-cycle infectivity assay was used to measure the neutralization of luciferase-encoding virions pseudotyped expressing the trimeric form of the S glycoprotein of SARS-CoV-2, as described previously (7). Supernatants containing SARS-CoV-2 pseudovirus were preincubated with serially diluted plasma from the three donors or serum from the patient at different time-points before and after plasma transfusions. After 1h at 37°C, all pre-incubated supernatants were added on ACE-2 transfected 293T cells and incubated for 72h at 37°C. The luciferase activity was measured using the EnVision. IC_{50} (serum dilution) was calculated by nonlinear regression analysis using the GraphPad Prism 8.3 software.

SARS-CoV-2 viral cultures

For assessment of infectious virus, VeroE6 cells were seeded at a density of $8x10^4$ cells/well in a 24 well plate and inoculated with 200 µl of viral transport medium the following day as reported in (8). Cells were inoculated for 1h at 37°C, then inoculum was removed and cells were further grown in regular cell medium. Cells were observed on day 3 and 7 or the presence of cytopathic effect (CPE) by light microscopy. In addition, supernatant was harvested immediately after inoculation and at each follow-up time-point. Isolation of replication competent SARS-CoV-2 was confirmed by presence of a CPE and an increase in viral RNA between consecutive time-point (8).

Safety and therapeutic outcome evaluation

The clinical symptoms were recorded by attending nurses and physicians daily. Following convalescent plasma transfusion, baseline laboratory exams (renal and hepatic function, complete blood count), biochemical (CRP and ferritin), lymphocyte enumeration (CD4, CD8 and B cell count) and antibody titers (total IgG, IgA and IgM, as well as anti-SARS-CoV-2 IgG and IgA) were evaluated every 2 to 7 days, while SARS-CoV-2 RNA was controlled by nasopharyngeal swabs every 2-3 days. Surveillance of plasma transfusion related adverse events and all severe adverse events was performed until day 7 after each transfusion. Chest CT scan was performed regularly before and after plasma transfusion as following; day 1, day 27, day 44, day 71, day 80 (post 1st cycle of plasma transfusion) and day 101 (post 3rd cycle of plasma transfusion). The primary outcome was the improvement in symptoms, biological parameters and chest CT scan lesions. The secondary outcome was the evaluation of isolation measures.

SUPPLEMENTARY FIGURE LEGENDS

SUPPLEMENTARY FIGURE 1. Measures of total Ig antibodies and SARS-CoV-2 RNA. **A,** Timeline showing total IgG, IgA and IgM antibody levels. **B,** High and stable viral loads were detected from nasopharyngeal swabs before the start of plasma transfusions. **C,** Strong detection of SARS-CoV-2 RNA in broncho-alveolar lavage and bronchial aspiration (at day 37 of diagnosis), but only at low viral loads in stools (at day 30) or undetectable in anal smears. **A and C,** The arrows indicate the 4 cycles of plasma transfusion (two units given on two consecutive days of each cycle).

SUPPLEMENTARY FIGURE 2. Timeline showing chest computed tomography (CT) scan before and after plasma transfusions. Representative axial CT image at day 27, day 44, and day 71 corresponding to time-points before the start of plasma transfusions, compared to day 80 and day 101, following the 1st and the 3rd cycle of plasma transfusion, respectively. Axial slices focused at the level of the anterior segment of the left upper lobe. Peribronchial ground- glass opacity and alveolar consolidation (blue arrow) was only observed on the second follow-up and subsequently disappeared. Ill-defined areas of ground glass opacities (orange arrows) were seen on the next CTs in other locations that disappeared at the final follow-up.

SUPPLEMENTARY FIGURE 3. Detection of anti-SARS-CoV-2 antibodies. **A,** Four cycles of ABO-identical plasma transfusion (two units on two consecutive days of each cycle) obtained from three COVID-19 convalescent donors were given at an interval of 10 to 15 days. These donors were selected based on the quantitative measures of anti-SARS-CoV-2 IgG antibody levels against the spike (S1) protein with EUROimmun ELISA (expressed as S/CO; signal to cut-off ratios), performed on their serum prior apheresis. n.a., not applicable. **B,** Quantitative anti-SARS-CoV-2 IgG antibody titers (in mcg/ml) as assessed by a Luminex assay for each plasma and in the patient's serum before and following plasma transfusions. **C,** Neutralization of SARS-CoV-2 pseudovirus by plasma from the three donors (top panel) and by sera from the patient's before and following plasma transfusions (bottom panel). The mean and SEM of three technical replicated are depicted. IC50 curve of neutralizing antibody activity. The dotted line indicates 50% neutralization. **D**, Timeline, including patient's follow-up after discharge (>day 115), showing absolute B cell counts (left panel) and anti-SARS-CoV-2 S protein IgG antibody levels (right panel).

SUPPLEMENTARY FIGURE 4. Emergence of a T-cell based immune response after plasma transfusions. A-E, Timeline showing total T cell counts (**A**), absolute counts of CD8 T cell subsets (naïve, central-memory, effector-memory, EMRA) (**B**), absolute counts of recent thymic emigrants and of CD4 T cell subsets (naïve, memory, CCR7+, CCR7-) (**C**), absolute counts of regulatory CD4 T cells (T reg) and of CD4 PD-1+ T cells (**D**), and of monocyte counts (**E**). **A-E,** The arrows indicate the 4 cycles of plasma transfusion (two units given on two consecutive days of each cycle); T cell subsets were assessed by CyTOF multi-parametric mass cytometry (9). **F,** B cell phenotype (CD27 and IgD) performed by CyTOF (9) at day 121 and day 203, when total B cell counts reached 17 cell/mm^3 and 253 cell/mm^3, respectively.

SUPPLEMENTARY REFERENCES

1. Greub G, Sahli R, Brouillet R, and Jaton K. Ten years of R&D and full automation in molecular diagnosis. *Future Microbiol.* 2016;11(3):403-25.
2. Opota O, Brouillet R, Greub G, and Jaton K. Comparison of SARS-CoV-2 RT-PCR on a high-throughput molecular diagnostic platform and the cobas SARS-CoV-2 test for the diagnostic of COVID-19 on various clinical samples. *Pathog Dis.* 2020;78(8).
3. Jacot D, Greub G, Jaton K, and Opota O. Viral load of SARS-CoV-2 across patients and compared to other respiratory viruses. *Microbes Infect.* 2020;22(10):617-21.
4. Harvala H, Robb ML, Watkins N, Ijaz S, Dicks S, Patel M, et al. Convalescent plasma therapy for the treatment of patients with COVID-19: Assessment of methods available for antibody detection and their correlation with neutralising antibody levels. *Transfus Med.* 2020.
5. Harvala H, Mehew J, Robb ML, Ijaz S, Dicks S, Patel M, et al. Convalescent plasma treatment for SARS-CoV-2 infection: analysis of the first 436 donors in England, 22 April to 12 May 2020. *Euro Surveill.* 2020;25(28).
6. Fenwick C, Croxatto A, Coste AT, Pojer F, Andre C, Pellaton C, et al. Changes in SARS-CoV-2 Spike versus Nucleoprotein Antibody Responses Impact the Estimates of Infections in Population-Based Seroprevalence Studies. *J Virol.* 2021;95(3).
7. Nie J, Li Q, Wu J, Zhao C, Hao H, Liu H, et al. Establishment and validation of a pseudovirus neutralization assay for SARS-CoV-2. *Emerg Microbes Infect.* 2020;9(1):680-6.
8. L'Huillier AG, Torriani G, Pigny F, Kaiser L, and Eckerle I. Culture-Competent SARS-CoV-2 in Nasopharynx of Symptomatic Neonates, Children, and Adolescents. *Emerg Infect Dis.* 2020;26(10).
9. Leipold MD, Obermoser G, Fenwick C, Kleinstuber K, Rashidi N, McNevin JP, et al. Comparison of CyTOF assays across sites: Results of a six-center pilot study. *J Immunol Methods.* 2018;453:37-43.
