# Supplementary material for: Case Report: Stepwise Anti-Inflammatory and Anti-SARS-CoV-2 Effects Following Convalescent Plasma Therapy With Full Clinical Recovery
Source: Front Immunol. 2021 Apr 21;12:613502. doi: 10.3389/fimmu.2021.613502 (PMC8097002; doi:10.3389/fimmu.2021.613502)
Supplement: Supplementary file 2 [file DataSheet_2.pdf]

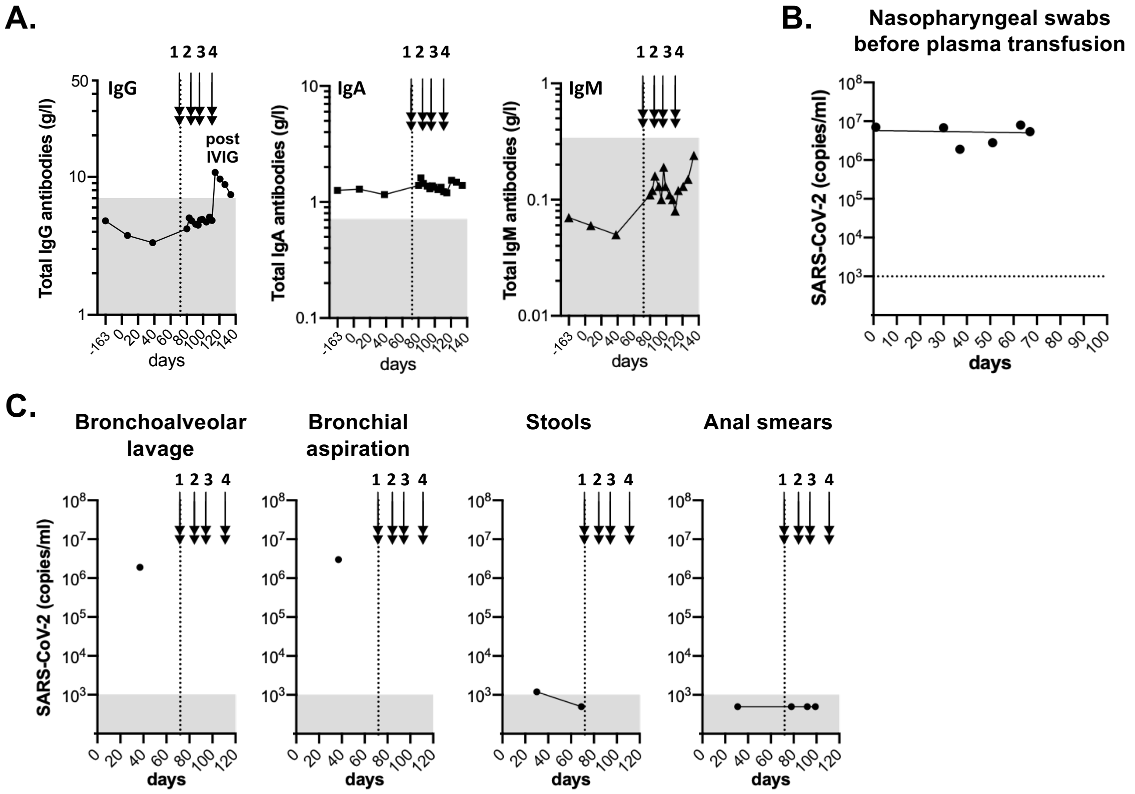

Supplemental Figure 1, Zimmerli et al.

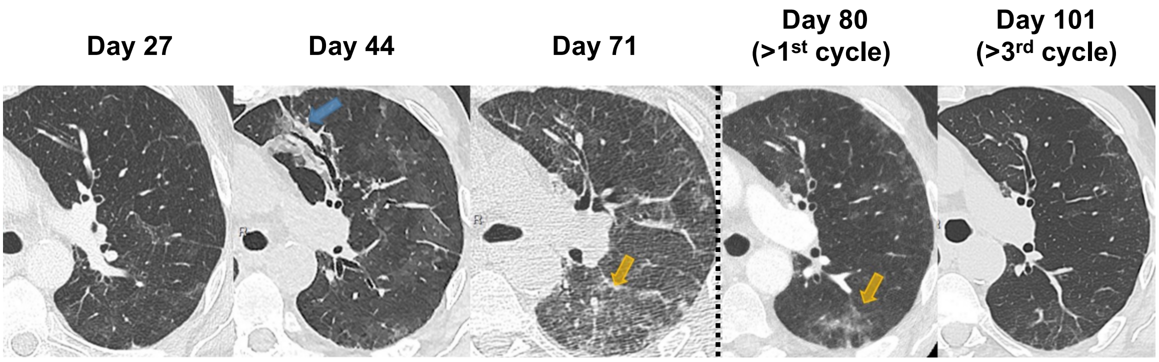

Supplemental Figure 2, Zimmerli et al.

A.

Date	Day	Cycles of plasma transfusion: Cycles	Transfused unit	Volume	EUROimmun/ELISA: anti-S1 IgG S/CO	anti-S1 IgA S/CO	Total IgG within plasma: g/l	g/unit of transfusion
11.06	72	Cycle 1	Donor 1 - unit 1	1x200 ml	4.43	1.62	10.3	2.06
12.06	73	Cycle 1	Donor 1 - unit 2	1x200 ml				2.06
23.06	84	Cycle 2	Donor 1 - unit 3	1x200 ml				2.06
24.06	85	Cycle 2	Donor 2 - unit 1	1x200 ml	5.29	2.95	8.23	1.65
3.07	94	Cycle 3	Donor 2 - unit 2	1x200 ml				1.65
4.07	95	Cycle 3	Donor 2 - unit 3	1x200 ml				1.65
20.07	111	Cycle 4	Donor 3 - unit 1	1x200 ml	7.67	n.a.	6.6	1.32
21.07	112	Cycle 4	Donor 3 - unit 2	1x200 ml				1.32

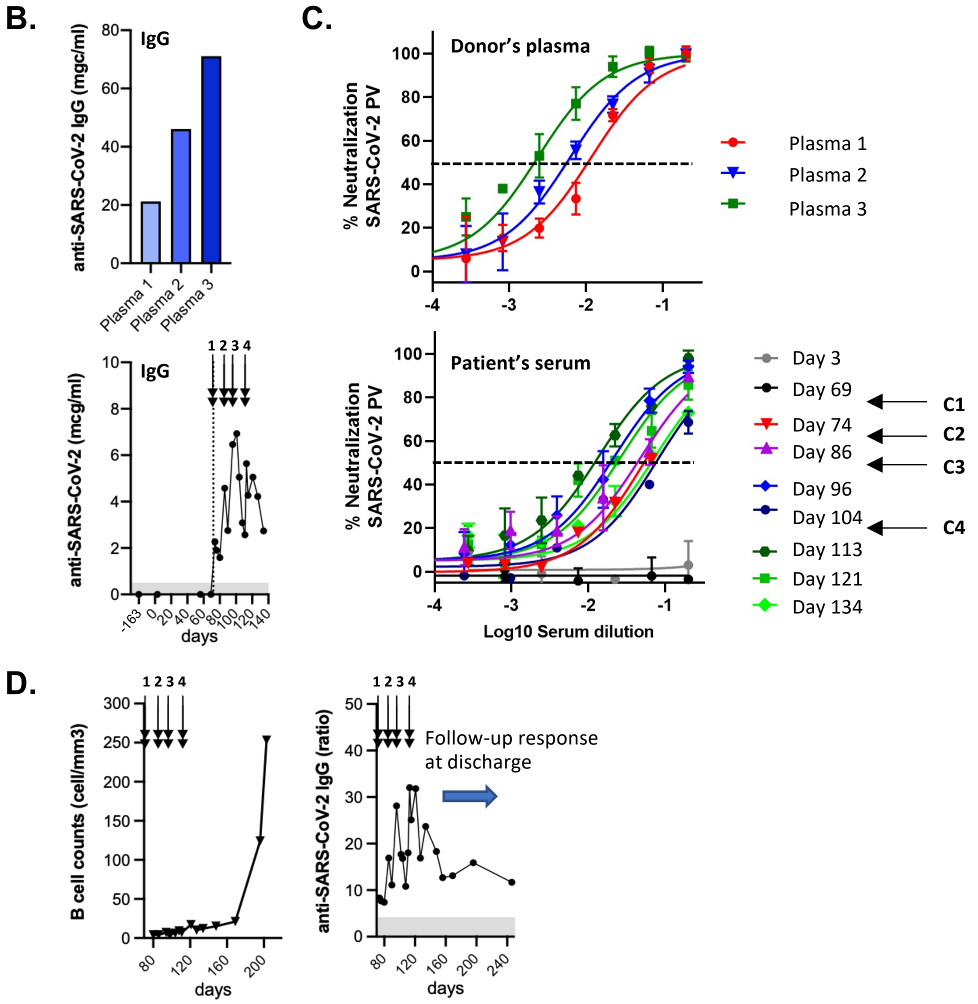

Supplemental Figure 3, Zimmerli et al.

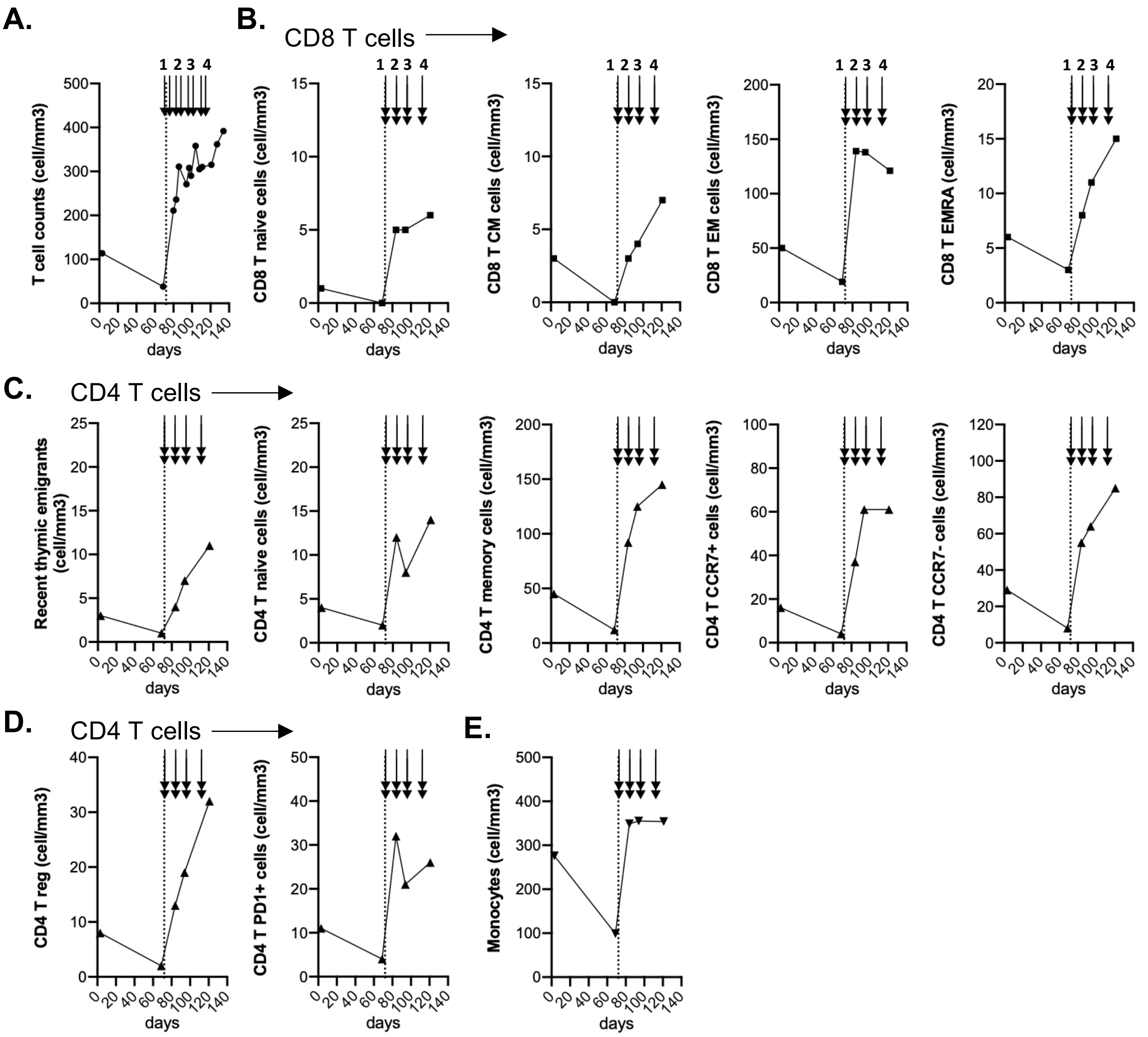

F.

Day 121 30.07.2020	Naive B cells	Unswitched memory B cells	Switched memory B cells	Late memory B cells
	CD27-IgD+	CD27+IgD+	CD27+IgD-	CD27-IgD-
cell/mm3	2	14	1	0
%	12.7	80.5	4.8	0
**Day 203 20.10.2020**	**Naive B cells**	**Unswitched memory B cells**	**Switched memory B cells**	**Late memory B cells**
	CD27-IgD+	CD27+IgD+	CD27+IgD-	CD27-IgD-
cell/mm3	157	3	1	49
%	61.8	1.3	17.6	19.3

Supplemental Figure 4, Zimmerli et al.
